# Development of a Measure of Intergenerational Contact: Implications for Evaluating Intergenerational Programs

**DOI:** 10.1093/geroni/igaa057.1765

**Published:** 2020-12-16

**Authors:** Shelbie Turner, Shannon Jarrott, Nicole Mullican, Karen Hooker

**Affiliations:** 1 Oregon State University, Corvallis, Oregon, United States; 2 The Ohio State University, Columbus, Ohio, United States; 3 BSS, Corvallis, Oregon, United States

## Abstract

Evaluating intergenerational programs often involves measuring the extent to which programs actually strengthen both the quantity and quality of intergenerational contact, which is associated with attitudes towards aging, social connection, and anxiety about aging. However, a surprisingly limited number of reliable, valid measures of intergenerational contact exist. Available measures are either explicit to familial intergenerational contact, are environment specific (e.g. workplace intergenerational contact), or are un-validated. In our presentation we will describe progress on our team’s efforts to develop an expert-informed reliable, valid measure of intergenerational contact that can be used widely to evaluate intergenerational programs. Specifically, we will share findings from a Delphi-style expert panel review used to develop the measure. The panel – 14 intergenerational research and practice experts – iteratively reviewed the measure, offering feedback on improving its relevance and practicality. We close with examples of how the measure can be used to evaluate intergenerational programs. Part of a symposium sponsored by Intergenerational Learning, Research, and Community Engagement Interest Group.

